# Chunks, Schemata, and Retrieval Structures: Past and Current Computational Models

**DOI:** 10.3389/fpsyg.2015.01785

**Published:** 2015-11-24

**Authors:** Fernand Gobet, Peter C. R. Lane, Martyn Lloyd-Kelly

**Affiliations:** ^1^Department of Psychological Sciences, University of LiverpoolLiverpool, UK; ^2^School of Computer Science, College Lane, University of HertfordshireHatfield, UK

**Keywords:** CHREST, chunk, long-term working-memory, modeling, retrieval structure, schema, theory

A recurring question in psychology and cognitive science concerns the expression of theories that are internally consistent and testable. Natural language is unsatisfactory, as theoretical concepts and mechanisms are not stated with sufficient precision (e.g., Newell et al., 1958; Newell and Simon, 1972; Farrell and Lewandowsky, 2010; Jones et al., 2014). Formal and, in particular, computational models avoid the problems of vagueness and under-specification by defining the processes and cognitive mechanisms that occur during a task. They additionally make quantitative and testable predictions, not only about the link between input and output, but also about fine-grained measures such as response times and eye movements. Further, such models can perform complex tasks and, when simulating learning, can use the statistical structure of the environment to help explain behavior.

This Opinion article briefly reviews the extent to which computational modeling has been used to develop theories accounting for the learning and use of chunks, schemata, and retrieval structures. We use the following definitions. A *chunk* is a “meaningful unit of information built from smaller pieces of information” (Gobet and Lane, 2012, p. 541), with the qualification that this information should be of the same kind. A *schema* is “a cognitive structure for representing and retrieving classes of typical situations for which a similar response is required of the learner” (Lane et al., 2000, p. 776). Finally, a retrieval structure is “a set of retrieval cues [that] are organized in a stable structure” (Ericsson and Kintsch, 1995, p. 216). We should point out that there exist plenty of definitions for these terms, which is actually an issue for progress in our understanding. For example, Richman et al. (1991) consider that a retrieval structure is a schema in long-term memory. Even fuzzier is the concept of a “chunk.” For example, a chunk is a unit of *declarative* memory in ACT-R (Anderson et al., 2004) and a unit of *procedural* memory in Soar (Newell, 1990), with none of the two meanings corresponding to the definition provided above. For a discussion of the multiple meanings of this term, see Gobet et al. (in revision).

## The perils of using verbal theories

To illustrate the weaknesses of verbal theories, let us consider how Ericsson and Kintsch (1995) applied their long-term working memory (LT-WM) theory to explain the results of Saariluoma's (1989) dictation task. Here, the experimenter dictated the content of a chess position, piece-by-piece, at a rapid rate (typically, one piece every 2 s), and then participants had to reconstruct the position. Saariluoma found that chess experts memorized the board positions taken from games fairly well (more than 80%), but obtained weaker performance with random positions (no more than 60%). Ericsson and Kintsch (1995) explained these results by proposing that strong chess players have acquired a hierarchical retrieval structure corresponding to the 64 squares of a chess board. This structure has two functions: first, it connects individual pieces to their respective squares, and, second, it allows pieces to be related to each other, representing a position as an integrated hierarchical structure. Thanks to this structure, it is possible to encode information rapidly into long-term memory, and to link it with patterns and schemata.

At first sight, this explanation looks plausible. However, when LT-WM was implemented as a computational model, Gobet (2000) found that recall ranged from 10 to 100% for both game and random positions, depending on parameter settings—essentially all possible outcomes. Being less specified with respect to mechanisms and parameters, LT-WM predicts even more possible outcomes, and is thus non-refutable, a serious problem for a scientific theory.

Of course, it could be the case that Gobet (2000) made incorrect theoretical choices in implementing LT-WM, in the sense that his model did not correspond to what Ericsson and Kintsch (1995) had in mind. Specifically, there exists an indefinite number of possible models that satisfy the verbal description they provided, and some may provide better results than the model implemented by Gobet (2000). However, this is precisely the point made by the authors highlighting the advantages of computational modeling: not enough constraints are provided by verbal theories, and thus too much freedom is left in the way they can be interpreted.

## A brief review of computer models

### Chunks

Although dominated by verbal theories, the literature includes a number of computational models of chunking. Several models have accounted for expert chess memory and perception (Ellis, 1973; Simon and Gilmartin, 1973; De Groot et al., 1996; Gobet and Simon, 1996a,b; Saariluoma and Laine, 2001) and the use of chunks for chess problem solving (Berliner and Campbell, 1984; Gobet and Jansen, 1994). Implicit learning has led to the development of several models (Servan-Schreiber and Anderson, 1990; French et al., 2011; Lane and Gobet, 2012a; Perruchet et al., 2014). Models have also been developed to account for short-term memory experiments (Robinet et al., 2011; Mathy and Feldman, 2012), spelling (Simon and Simon, 1973), alphabet recitation (Klahr et al., 1983), and verbal learning (Feigenbaum and Simon, 1984; Gobet et al., 2001). Mathematical models have been developed to estimate the most efficient size of chunks (Dirlam, 1972), the number of chunks necessary to reach expertise (Simon and Gilmartin, 1973), or the amount of monochrestic (single-use) knowledge held by experts (Chassy and Gobet, 2011). Rabinovich et al. (2014) used non-linear dynamics to develop a model of hierarchical chunking in the brain.

Some of these models have led to important empirical discoveries. For example, modeling with CHREST (Gobet and Simon, 1996a,b) predicted that, contrary to a widely held opinion at the time, chess experts should show superior memory recall over weaker players even for random positions. To explain this counter-intuitive prediction, Gobet and Simon argued that a model with a larger number of chunks was more likely to find, serendipitously, chunks in a random position than a model with few chunks. The prediction was supported by a meta-analysis of the available data as well as the collection of new data. Interestingly, Vicente and Wang (1998) challenged this explanation by arguing that the chess positions used in the literature still contain constraints (e.g., there is only one white King and at most eight black pawns). They argued that, with positions where all pieces have the same likelihood of being selected (“truly random positions”), the skill effect should disappear. By contrast, simulations with CHREST predicted that, again, there should be a reliable skill effect, albeit smaller than with standard random positions, because the likelihood to find a chunk fortuitously is smaller. The model's predictions were upheld (Gobet and Waters, 2003): not only did the empirical data show a skill effect, but they also were close to the absolute values predicted by the model. This result is theoretically important, because the other mechanisms proposed to explain experts' superiority with game positions (e.g., high-level knowledge, schemata, or retrieval structures) fail to explain their superiority with random and fully random positions (Gobet, 2015). It thus provides direct support for chunking mechanisms. In addition, and importantly, the fact that new predictions were made by a computational model and supported empirically is important, since this rebuts a common criticism that computational modeling only serves to fit data.

### Schemata

Unfortunately, there has been little modeling work with respect to schemata (Lane et al., 2000). CHREST is perhaps unique in explaining the processes underpinning the learning and use of schemata (called *templates*) in a variety of domains. Simulations have been carried out in board games (Gobet and Simon, 2000; Gobet, 2009; Bossomaier et al., 2012), diagrammatic reasoning (Lane et al., 2001), implicit learning (Lane and Gobet, 2012a), categorisation (Lane and Gobet, 2014), and agent modeling (Lloyd-Kelly et al., 2014, 2015). When a broader definition of a schema is used, work with neural networks (St. John and McClelland, 1990) and Soar (Laird et al., 1987) address issues related to schemata; Kintsch (1992) proposes some simulations with respect to language.

### Retrieval structures

Overall, the least modeling research has been carried out on retrieval structures. To our knowledge, the only models having tackled this issue used *chunking networks*. Richman et al. (1995) used EPAM-IV to simulate the growth of expertise in the digit-span task, accounting for how an individual was able to memorize up to 106 digits dictated every second. The retrieval structure used in their simulations consisted of digits at the bottom level, chunks (e.g., running times) at the second level, super-groups combining chunks at the third level, and super-group clusters combining super-groups at the fourth level. Gobet (2013) used CHREST to simulate how a chess master was able to memorize several briefly presented chess boards simultaneously. In this model, the retrieval structure consisted of the list of chess world champions. In both cases, the retrieval structures were directly based on the strategies used by the human participants.

## Conclusion

While a fair number of models have investigated chunking, very little computational work has been devoted to schemata and retrieval structures, although some informal theories exist. This is regrettable, since schemata and retrieval structures are key structures of the human mind, not least because they link together various kinds of knowledge. Why are there so few models? Possible answers include the lack of methodology for developing models (but see Lane and Gobet, 2012b), the technical skills and time required, the difficulty of deciding what constitutes a good model (Roberts and Pashler, 2000), the poor specification of current theories, and also the difficulty in finding suitable data to model. Naively, one could argue that modeling is not necessary, as data from neuroscience will answer all the key questions in the long term. However, this is unlikely to be the case. Data from neuroscience are actually inconsistent and confusing (Uttal, 2011; Guida et al., 2012) and computational models are necessary for making sense of them (Gobet, 2014)!

A striking result of this review is the number and breadth of coverage of models for the chunking network family, whose main representatives are EPAM and CHREST. This should not really come as a surprise and reflects the influence of Herbert Simon, who was one of the first to advocate chunking as a key mechanism of human cognition (e.g., Feigenbaum and Simon, 1962; Simon, 1974) and also carried out influential empirical research on retrieval structures and schemata (Larkin et al., 1980; Gobet and Simon, 1996c). He also studied topics, most notably expertise in chess, for which chunking comes as a natural explanation (e.g., Simon and Chase, 1973). Finally, chunks, schemata, and retrieval structures dovetail with his hypothesis that human cognition can be described as a physical symbol system (Simon and Newell, 1976), where symbols are discrete units.

Beyond historical reasons, chunking network models offer several theoretical advantages, including: (a) they provide learning mechanisms; (b) they are organized hierarchically; (c) they include time parameters, making it possible to make precise predictions; (d) they are efficient and scale up (learning hundreds of thousands of chunks can be done in a few minutes; (e) they provide an architecture seamlessly implementing chunks, schemata and retrieval structures; and (f) they can be (and have been) used to account for phenomena in different psychological provinces, including perception, memory, problem solving and decision making, and language.

Without the use of formal models, in particular computational models, progress in understanding chunks, retrieval structures, and schemata will be slow, if possible at all. By highlighting the strengths of computational modeling and the weaknesses of verbal theorizing, we hope to have encouraged other researchers to develop computational models accounting for each of these structures and how they work together.

## Funding

FG is a Professor in the International Centre for Language and Communicative Development (LuCiD) at the University of Liverpool. The support of the Economic and Social Research Council [ES/L008955/1] is gratefully acknowledged.

### Conflict of interest statement

The authors declare that the research was conducted in the absence of any commercial or financial relationships that could be construed as a potential conflict of interest.
